# Alternative splicing of human telomerase reverse transcriptase in gliomas and its modulation mediated by CX-5461

**DOI:** 10.1186/s13046-018-0749-8

**Published:** 2018-04-10

**Authors:** Guihong Li, Jing Shen, Junguo Cao, Guangtong Zhou, Ting Lei, Yuxue Sun, Haijun Gao, Yaonan Ding, Weidong Xu, Zhixin Zhan, Yong Chen, Haiyan Huang

**Affiliations:** 1 Department of Neurosurgery, First Hospital of Jilin University, Changchun, 130021 China; 2 Department of Neurosurgery, First Affiliated Hospital of Zhengzhou University, Zhengzhou, 450000 China; 3 Department of Cardiology, Shengze Hospital of Jiangsu Province, Suzhou, 215200 China; 4 Department of Neurovascular Research Laboratory and Neuroscience, Universitat Autonoma de Barcelona, 08035 Barcelona, Spain

**Keywords:** Glioma, Human telomerase reverse transcriptase (hTERT), Alternative splicing, CX-5461, G-quadruplex

## Abstract

**Background:**

Glioma is a heterogeneous, invasive primary brain tumor with a wide range of patient survival and a lack of reliable prognostic biomarkers. Human telomerase reverse transcriptase (hTERT) has been reported in the presence of multiple transcripts in various tumor systems. The biological function and precise regulatory mechanisms of hTERT transcripts remain uncertain.

**Methods:**

Alternative splicing of hTERT and telomerase activity were examined in 96 glioma specimens, including 38 glioblastomas (GBMs), 23 oligodendrogliomas (ODMs), and 35 oligoastrocytomas (OAMs). The correlation between telomerase activity or hTERT transcripts and patient clinical characteristics was investigated. We examined the regulation of alternative splicing of hTERT and telomerase activity by G-quadruplex stabilizer CX-5461 in GBM cells. The biological effects of CX-5461 on GBM cell lines, including inhibition of cell proliferation, effects on cell cycle/apoptosis, and telomere DNA damage were further explored.

**Results:**

The β splicing was verified in human gliomas and hTERT+β was significantly correlated with higher telomerase activity, higher KPS, larger tumor size, and higher tumor grades. Meanwhile, glioma patients lacking hTERT+β expression or telomerase activity showed a significant survival benefit. Notably, CX-5461 altered hTERT splicing patterns, leading to an increase of hTERT-β transcript and a decrease of hTERT+β transcript expression, which inhibits telomerase activity. In addition, CX-5461 had cytotoxic effects on GBM cells and caused telomere DNA damage response, induced G2/M arrest and apoptosis.

**Conclusions:**

The hTERT+β is verified to be correlated with clinical parameters in gliomas, and could serve as a prognostic marker or possibly therapeutic target for gliomas. CX-5461 can regulate the splicing pattern of hTERT, inhibit telomerase activity, and kill GBM cells.

## Background

**Fig. 1:**
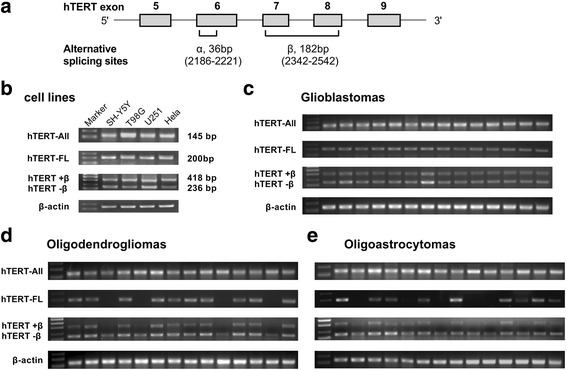
hTERT alternative splice variant patterns in human gliomas and cell lines. **a** Two main alternative splicing sites: the α splice site in exon 6, which produces a 36-bp in frame deletion; and the β splice site in exon 7 and exon 8, which results in a 182-bp deletion and nonsense mutation that truncates the protein. hTERT variant patterns of cell lines (**b**) and human gliomas including 38 GBMs (**c**), 24 ODMs (**d**) and 35 OAMs (**e**) were investigated by RT-PCR (Only a representative portion of the total analyzed samples was shown in the gels). Two hTERT alternative splice variants (hTERT+β and hTERT–β) were detected in subsets of the tumors and cell lines. Thirty-three of 38 (86.8%) GBMs, 17 of 24 (73.9%) ODMs and 15 of 35 (42.9%) OAMs exhibited hTERT+β transcript, and the remaining 31 cases only exhibited hTERT–β transcript. Accordingly, cases with hTERT +β transcript were consistent with the expression of hTERT-FL transcript

Telomeres are essential to maintain the stability of chromosome ends, and two telomere maintenance mechanisms are known [1]. The most frequent one is telomerase, a specific enzyme that adds telomeric repeats to chromosomes and lengthens telomeres. Most cancer cells escape telomere shortening by activating the telomerase, which leads to unlimited proliferation capacity and immortalization. About 10% of cancer cells do not show telomerase activity, but instead maintain telomere length by the recombination-based alternative lengthening of telomeres (ALT) mechanism [1–3]. Telomerase is a ribonucleoprotein with two key subunits: human telomerase RNA (hTR), which acts as a transcription template [4, 5], and human telomerase reverse transcriptase (hTERT), whose expression controls enzymatic activity [6]. In humans, the hTR transcript is constitutively produced, whereas the production of hTERT is highly regulated at both the transcriptional levels and post-transcriptional levels [7–9]. A number of studies have shown the presence of different transcripts of hTERT, including three deletions and four insertions, which may affect telomerase activity and biological functions [10–12]. Further evidence has accumulated that only a full-length hTERT (hTERT-FL) transcript is associated with telomerase activity. Four insertions and the β and γ deletion result in nonfunctional proteins whereas the α deletion is a dominant negative inhibitor of telomerase activity [13, 14]. The most widely studied variants involve splicing at two main sites: the α splice site in exon 6, which produces a 36-bp in frame deletion within the conserved reverse transcript motif A; and the β splice site in exon 7 and exon 8, which results in a 182-bp deletion and nonsense mutation that truncates the protein [15–18] (Fig. 1a). Alternative splicing of hTERT mRNA has been shown to contribute to the regulation of telomerase activity and might be used as an additional prognostic marker in certain types of malignancies.

Gliomas are the most frequent primary brain tumors, characterized by high aggressiveness based on local diffuse infiltration, and always remain poor prognosis [19]. Gliomas show marked heterogeneity at the microscopic and molecular levels, which are mainly reflected in the clinical course of individual changes. However, only a few reliable biomarkers currently exist to predict the course of disease or outcome of glioma patients [20]. Studies show that high telomerase activity or hTERT promoter mutations are prognostic markers for human brain tumors [21–25]. In association with tumor histopathology and clinical parameters, expression of hTERT transcripts may serve as diagnostic or prognostic biomarkers for certain cancer patients [17, 18, 26].

Telomerase is an attractive target for cancer treatment, and the regulation of hTERT alternative splicing transcript patterns would be one therapeutic strategy [27]. Telomerase activity decreases with down-regulation of active hTERT mRNA transcription. Previous studies have shown that transforming growth factor-β (TGF-β) and antisense oligonucleotides treatments inhibit telomerase activity by altering the splicing patterns of hTERT pre-mRNA [28, 29]. Another study reported a G-quadruplex stabilizer called 12,459 that converts hTERT-FL to hTERT-β transcript in A549 cells [30]. G-quadruplex structures have been extensively studied in the telomeric single-stranded overhang or c-myc gene promotor sequence, and hTERT intron 6 was reported to have two G-rich sequences able to form G-quadruplex, which may affect β splicing [30–32]. Similarly, CX-5461, as an rDNA transcription inhibitor currently in phase I trials for malignancies, is reported to strongly bind and stabilize G-quadruplex and induce DNA damage in vitro [33–35]. In addition, other G-quadruplex stabilizers, BRACO-19 and TMPyP4, also have the potential to regulate alternative splicing variants of hTERT [36, 37].

In this study, we investigated hTERT alternative splice variant patterns of human gliomas and its correlation with telomere activity. Then, we focused on the correlation between telomerase activity or hTERT-FL mRNA and patient clinical characteristics.

We examined the regulation of alternative splicing of hTERT and telomerase activity by G-quadruplex stabilizer CX-5461 in GBM cells. The biological effects of CX-5461 on GBM cell lines, including inhibition of cell proliferation, effects on cell cycle/apoptosis, and telomere DNA damage were further explored.

## Methods

### Cell lines and cell culture

Six tumor cell lines, T98G (human glioblastoma), U251 (human glioblastoma), SH-SY5Y (human neuroblastoma), Hela (human cervical cancer), GM847 (ALT+, TA-, human fibroblast), C6 (rat glioma), and one normal cell line HEB (Human normal astrocyte) were obtained from the Shanghai Institute of Cell Biology at the Chinese Academy of Sciences (Shanghai, China). The cells were grown in Dulbecco’s modified eagle medium (DMEM) supplemented with 10% fetal bovine serum (FBS) (Biowest, South America Origin) and antibiotics (100 units/mL penicillin and streptomycin) in a humidified incubator maintained at 37 °C with 95% air and 5% CO_2_. Cells at logarithmically growing phases were harvested for hTERT mRNA and telomerase activity analyses. Hela cell line was regarded as positive control for telomerase activity analyses and GM847 (ALT+) cell line was regarded as negative control for telomerase activity analyses. CX-5461 compound was purchased from Selleck Chemicals. TMPyP4 and BRACO-19 were purchased from R&D Systems.

### Patients and tissue specimens

The cohort consisted of 96 patients and diagnosed between 2012 and 2014 from the first hospital of Jilin University. All patients with glioma and corresponding clinical information were obtained with consent and the project was approved by the ethics committee of the First Hospital of Jilin University. The details concerning age, gender, KPS, tumor size (diameter), histopathology and WHO grade, and extent of resection were retrospectively summarized in Table 1. Samples included 38 glioblastomas (GBMs, Grade IV), 23 oligodendrogliomas (ODMs, Grade II or III), and 35 oligoastrocytomas (OAMs, Grade II or III). After surgery, samples were snap frozen in liquid nitrogen and stored at − 80 °C until use.

**Table 1:** Characteristics of glioma patients according to TA and hTERT transcript

Characteristics	*n* patients (%)	TA, *n* patients (%)		*P*	hTERT, *n* patients (%)		*P*
	*n* = 96	Positive 67 (69.8)	Negative 29 (30.2)		hTERT +β 65 (67.7)	hTERT -β 31 (32.3)	
Gender				1			1
Male	51 (53.1)	36 (70.6)	15 (29.4)		35 (68.6)	16 (31.4)	
female	45 (46.9)	31 (68.9)	14 (31.1)		30 (66.7)	15 (33.3)	
Age				0.007*			0.029*
<50	52 (54.2)	30 (57.7)	22 (42.3)		30 (57.7)	22 (42.3)	
≥ 50	44 (45.8)	37 (84.1)	7 (15.9)		35 (79.5)	9 (20.5)	
KPS				0.012*			0.015*
<75	57 (59.4)	34 (59.6)	23 (40.4)		33 (57.9)	24 (42.1)	
≥ 75	39 (40.6)	33 (84.6)	6 (15.4)		32 (82.1)	7 (17.9)	
Tumor size (cm)				0.008*			0.002*
<6	49 (51)	28 (57.1)	21 (42.9)		26 (53.1)	23 (46.9)	
≥ 6	47 (49)	39 (83)	8 (17)		39 (83)	8 (17)	
Histopathology and WHO grade				0.001*			0.001*
GBMs (IV)	38 (39.6)	34 (89.5)	4 (10.5)		33 (86.8)	5 (13.2)	
ODMs (III)	23 (24)	17 (73.9)	6 (26.1)		17 (73.9)	6 (26.1)	
OAMs (III, II)	35 (36.4)	16 (45.7)	19 (54.2)		15 (42.9)	20 (57.1)	
Extent of resection				0.082			0.334
GTR	71 (74)	46 (64.8)	25 (35.2)		46 (64.8)	25 (35.2)	
STR	25 (26)	21 (84)	4 (16)		19 (76)	6 (24)	

*P-values indicating significance; *KPS* Karnofsky Performance Score, *GTR* Gross-total Resection; *STR* Subtotal Resection

### RNA extraction, reverse transcription, PCR

Total cellular RNA in the cell lines and tissue specimens were extracted using the EasyPure RNA kit (TRANSGEN BIOTECH). Reverse transcription was performed with 1 μg of total RNA and oligo (dT) primers by TransScript One-step gDNA Removal and cDNA Synthesis (TRANSGEN BIOTECH). The relative gene expression levels of hTERT alternative splice variants were analyzed by PCR using primers designed according to GenBank accession no. AF015950. The PCR primer sequences specific for all the variants of hTERT (hTERT-All) mRNA were 5’-CGGAAGAGTGTCTGGAGCAA-3′ (1784–1803, forward) and 5’-GGATGAAGCGGAGTCTGGA -3′ (1928–1910, reverse). The PCR primer sequences specific for hTERT-FL transcript were 5’-TGTACTTTGTCAAGGTGGATGTG-3′ (2172–2194, forward) and 5’-GTACGGCTGGAGGTCTGTCAAG-3′ (2371–2350, reverse). The primers set for hTERT α or β splicing transcript variant were 5’-CCGCCTGAGCTGTACTTTGTC-3′ (2162–2183, forward) and 5’-CAGAGCAGCGTGGAGAGGAT-3′ (2580–2560, reverse), which produced four possible products, α + β + (418 bp), α + β– (236 bp), α–β + (382 bp), and α–β– (200 bp), respectively. All PCR was performed in 50 μL of reaction mixture using 2 μL of the cDNA and Ex Taq DNA polymerase (TaKaRa) by incubation at 94 °C for 2 min, followed by 35 amplification cycles of 94 °C for 30 s, specific annealing temperature for 45 s, and 72 °C for 60 s, and a final extension at 72 °C for 5 min. Annealing temperature was 58 °C for hTERT 1784/1928, 63 °C for hTERT 2172/2371, and 62 °C for hTERT 2162/2580. Amplified products were electrophoresed on 2% agarose gels with GelStar Nucleic Acid Gel Stain (LONZA) or electrophoresed on a 12% nondenaturing polyacrylamide gel staining with 0.2% AgNO3. Images were photographed using a UVP gel documentation system (Ultraviolet Products, Upland, CA, USA). The expression of β-Actin or β2m was served as an internal control.

### Telomere repeat amplification protocol (TRAP) assay

For the assessment of telomerase activity (TA), a modified version of the telomere repeat amplification protocol (TRAP) assay was applied. Briefly, telomerase was prepared from extracts of 2 × 10^5^ exponentially growing cells or 40 mg tumor samples by lysing for 30 min on ice in 200 μL TRAPEZE® 1 × CHAPS Lysis Buffer (Millipore s7750). The lysate was then centrifuged at 12,000 g for 20 min at 4 °C, and the supernatant was collected, frozen in liquid nitrogen and stored at − 80 °C for use. Total cellular protein was then determined, we assayed 1 μg of protein extract in a 40 μL reaction mixture that contained 10 × TRAP buffer (4.0 μL), bovine serum albumin (BSA, 0.5 μL, 0.05 μg sample^− 1^), dNTPs mix (2.0 μL, 2.5 mM, TaKaRa), TS primer (1.0 μL, 100 ng μL ^− 1^), and DEPC (diethyl pyrocarbonate)-treated water (31.5 μL). Negative control involved incubating 1.0 μL of cell lysate at 94 °C for 10 min prior to primer extension. The Hela cell line (American Type Culture Collection) served as a positive control. Then the mixtures were involved incubation for 45 min at 30 °C for the initial elongation step, followed by 94 °C for 5 min. The elongated products were then subjected to PCR amplification. The PCR master mix consisted of 10 × TRAP buffer (1.0 μL), dNTPs mix (4.0 μL, 2.5 mM, TaKaRa), TS primer (1.0 μL,100 ng μL ^− 1^), primer mix (2.0 μL; ACX reverse primer 100 ng μL ^− 1^; NT primer 100 ng μL ^− 1^, and TSNT internal control primer 1× 10^− 7^ μM), Ex Taq polymerase (0.5 μL, 5 U μL ^-1^), DEPC water (1.5 μL) and elongated products (40 μL). The mixture was amplified at 94 °C for 2 min, followed by 35 cycles of PCR reaction (94 °C for 30 s, 52 °C for 30 s, and 72 °C for 60 s) on an AMPLITRON® Thermolyne (Alpha Multiservices, Inc). Amplified products were visualized on a 12% nondenaturing polyacrylamide gel, after electrophoresis and staining with 0.2% AgNO3. Images were photographed using a UVP gel documentation system (Ultraviolet Products, Upland, CA, USA). Telomerase activity was assessed by determining the ratio of the entire telomerase ladder to that of the internal control, using Lab works 4.5 image analysis software.

### Western blot

Cells in the log growth phase were seeded on 100 mm dishes (Falcon) and allowed to adhere overnight prior to treatment. After treatment for 48 h, cells were collected and lysed in RIPA buffer, and centrifuged at 12,000×g for 20 min. The total protein concentration was quantified with a bicinchoninic acid assay kit. Equal amounts of proteins were loaded onto SDS-PAGE gels, and separated proteins were transferred to PVDF membranes. After blocking with 5% skim milk, the membranes were incubated with primary antibodies against hTERT (Abcam, ab32020, diluted 1:1000), cyclin D1 (Cell Signaling Technology, #2978, diluted 1:1000), p53 (Cell Signaling Technology, #2527, diluted 1:1000), p16 (Abcam, ab81278, diluted 1:2000), γ-H2AX (Abcam, ab81299, diluted 1:5000), 53BP1 (Abcam, ab175933, diluted 1:5000), p-ATM (Abcam, ab81292, diluted 1:5000), Bcl-2 (Cell Signaling Technology, #4223, diluted 1:1000), caspase-3 (Cell Signaling Technology, #9662, diluted 1:1000) or Bax (Cell Signaling Technology, #5023, diluted 1:1000) and a monoclonal antibody against β-actin (Cell Signaling Technology, #4970, diluted 1:1000) was used as a protein endogenous control. The membranes were washed three times with PBS-T (0.1% [*v*/v] Triton-X 100) buffer for 0.5 h, then incubated with goat anti-rabbit IgG (H + L) poly-HRP secondary antibody (Thermo Fisher Scientific, #32260, diluted 1:10000) for 2 h. After washing with the PBS-T buffer again, membranes were scanned with the Odyssey Infrared Imaging System (LI-COR).

### Cell viability assay

The cytotoxicity of CX-5461 to GBM cells was determined by the MTT assay. T98G, U251, C6 and HEB cells were seeded in 96-well plate and exposed to CX-5461 at concentration of 0, 0.04, 0.2, 1, 5, 25 μM for 48 h. 20 μl MTT (5 mg/ml) (Promega, Shanghai, China) was added to each well and incubated for 4 h. Dimethyl Sulphoxide (DMSO) was added to the well after discarding the supernatant. Then, the plate was shaken for 3 min. The absorbance was measured at 570 nm wavelength using a Microplate Reader (Bio-Rad, Hercules, CA, USA).

### Colony formation assay

Cells in the log growth phase were seeded in 6-well plates at a density of 1 × 10^3^ cells per well and allowed to attach to the plate overnight prior to treatment. Cells were then incubated with CX-5461 at the indicated concentrations for 8 days. Then, they were fixed with 4% formaldehyde and stained with crystal violet. The number of colonies with at least 50 cells was counted under a microscope.

### Flow cytometric assays

Briefly, cells were seeded in 6-well plates at a density of 2 × 10^5^ cells per well. After treatment with CX-5461, cells were harvested with the accutase detachment solution (Sigma Aldrich, USA). For the detection of cell cycle, cells were fixed in 70% ethanol for 12 h at − 20 °C after collection, then washed twice with PBS, and incubated with 1 g/ml propidium iodide (PI) and RNase for 30 min. Apoptotic cells were quantified with an annexin-V-fluorescein isothiocyanate (FITC)/propidium iodide (PI) apoptosis detection kit (BD) according to the manufacturer’s instructions. Then the stained cells were analyzed with a flow cytometer. We used the FACSDiva Version 6.2 to calculate the numbers of viable cells (annexin-V–/PI–), early apoptotic cells (annexin-V+/PI–), and late apoptotic cells (annexin-V+/PI+).

### Statistical analysis

All experiments were performed in triplicate, unless otherwise noted. The analyzed results are expressed as the mean ± SD. Groups were compared with the unpaired Student’s t-tests. Survival curves were illustrated by Kaplan-Meier plots, and significance was calculated by log-rank test. Characteristics according to TA or hTERT transcript were evaluated by χ^2^ test. All analyses were performed with SPSS software version 21.0 for Windows or Graph Pad Prism 7.0.

## Results

### hTERT alternative splice variant patterns in human gliomas and cell lines

We investigated hTERT transcript patterns by RT-PCR, and found that hTERT-All expressed in all human glioma tissues and cell lines (Fig. 1b-e). Then, we used another pair of primers specific to hTERT-FL mRNA to perform PCR analysis on the same batch of cDNA. While hTERT-FL mRNA was detected in about 86.8% GBMs, 73.9% ODMs, and 42.9% OAMs (Fig. 1c-e). Considering the existence of different alternative splice variants, the expression of hTERT was further characterized. Two hTERT alternative splice variants (hTERT+β and hTERT–β) were detected in subsets of the gliomas, as illustrated for representative cases (Fig. 1c-e). However, α splice variant was not detected in any human glioma tissues or GBM cell lines. Thirty-three of the 38 GBM cases, 17 of the 23 ODMs and 15 of the 35 OAMs exhibited hTERT+β transcript, and the remaining 31 cases only exhibited hTERT–β transcript. Accordingly, cases with hTERT+β transcript were consistent with the expression of hTERT-FL transcript. When hTERT+β transcript expression was correlated with clinical variables, its presence was found to be significantly associated with age, KPS, tumor size, and WHO tumor grade (Table 1). These findings indicate that hTERT+β transcript is associated with more aggressive growth of gliomas.

### Concomitant of telomerase activity and hTERT+β transcript in human gliomas and cell lines

**Fig. 2:**
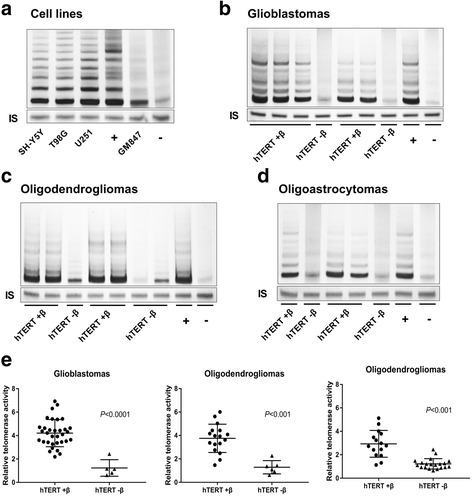
Relative telomerase activity in human gliomas and cell lines. **a** Telomerase activity was detected by TRAP assay, and all four cell lines exhibited high TA. Thirty-four of 38 (89.5%) GBMs (**b**), 17 of 23 (73.9%) ODMs (**c**), and 16 of 35 (35.7%) OAMs (**d**) exhibited high TA. **e** The high telomerase activity of human gliomas was significantly consistent with the expression of hTERT+β transcript

Relative telomerase activity of 96 glioma samples and cell lines was detected by TRAP assay. Thirty-four of 38 (89.5%) GBMs, 17 of 23 (73.9%) ODMs, and 16 of 35 (45.7%) OAMs and GBM cell lines exhibited high TA (Fig. 2a-d), which was consistent with the expression of hTERT-FL or hTERT+β transcript (Fig. 2e). We found that TA was not related to the patient’s gender or extent of resection. However, TA was significantly correlated with age, tumor size, KPS, and WHO grades (Table 1). Thirty of 52 (57.7%) patients with age < 50 were positive for TA, while 37 of 44 (84.1%) patients with age ≥ 50 were positive. Twenty-eight of 49 (57.1%) patients with glioma size < 6 cm were positive for TA, while 39 of 47(83%) with glioma size ≥6 cm were positive. And, thirty-four of 57 (59.6%) patients with KPS < 75 were positive for TA, while 33 of 39 (84.6%) patients with KPS ≥ 75 were positive. Expression of hTERT+β transcript was positively correlated with elevated telomerase activity, which may indicate deterioration of the disease.

### Telomerase status and hTERT+β transcript predict survival in human gliomas

**Fig. 3:**
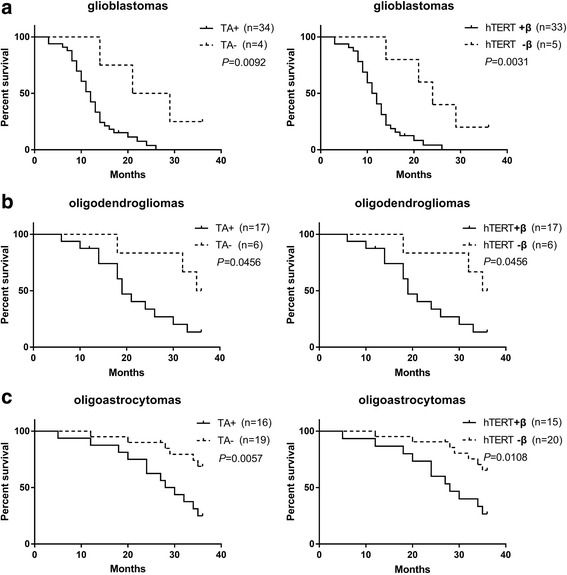
Telomerase-associated parameters compared with overall survival. Kaplan–Meier estimates revealed a significant survival benefit for glioma patients lacking hTERT+β (Right) or TA (Left) in different WHO grade gliomas including 38 GBMs (**a**), 23 ODMs (**b**) and 35 OAMs (**c**)

To evaluate the effects of telomerase activation on disease progression, after 36 months of follow-up, we correlated the expression of hTERT+β (hTERT-FL) or TA with the survival time of glioma patients. Kaplan–Meier estimates revealed a significant survival benefit for patients lacking hTERT+β or TA in different WHO grade gliomas including 38 GBMs, 23 ODMs and 35 OAMs (Fig. 3a-c). Hence, we find a correlation between clinical outcome and hTERT-FL transcript or high TA in glioma patients.

### CX-5461 could alter hTERT alternative splicing in GBM cells

**Fig. 4:**
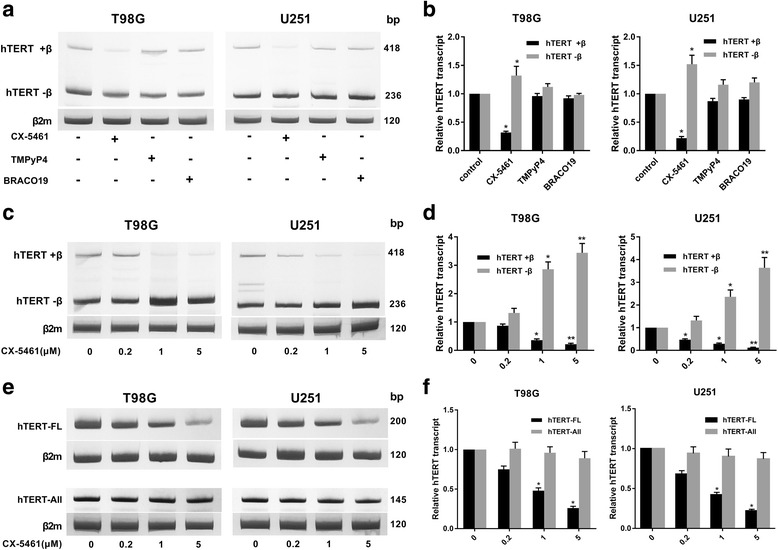
CX-5461 altered hTERT alternative splicing in GBM cells. **a** The expression of hTERT transcripts were investigated by RT-PCR analysis. CX-5461 (1 μM, 48 h) markedly altered its splicing patterns, leading to a sharp decrease of hTERT+β transcript in T98G and U251 cells. While TMPyP4 and BRACO-19, two other G-quadruplex stabilizers, did not significantly alter its splicing patterns. **b** The histogram showed transcripts expression levels relative to control. **c** CX-5461 could lead to a decrease of hTERT+β transcript and to an increase of hTERT-β in a dose-dependent manner in T98G and U251 cells. **d** The histogram showed transcripts expression levels relative to control. **e** CX-5461 could lead to a significant decrease of hTERT-FL in a dose-dependent manner and had no effect on hTERT-All. **f** The histogram showed transcripts expression levels relative to control. *P < 0.05, **P < 0.01 vs. control group

To explore the regulation of hTERT alternative splicing, the GBM cells were exposed to 3 different G-quadruplex stabilizers. The expression of hTERT transcripts were investigated by RT-PCR. CX-5461 significantly altered its splicing patterns resulting in a dramatic drop of hTERT+β transcript in T98G and U251 cells (Fig. 4a, b). While the other two G-quadruplex stabilizers, TMPyP4 and BRACO-19 (1 mM, 48 h) did not significantly alter its splicing patterns. The expression of alternative splicing of hTERT was detected after GBM cells were exposed to different concentrations of CX-5461 (0, 0.2, 1, 5 μM) for 48 h. As predicted, CX-5461 could lead to a decrease of hTERT+β transcript and to an increase of hTERT-β in a dose-dependent manner both in T98G and U251 cells (Fig. 4c, d). Similarly, CX-5461 could lead to a significant decrease of hTERT-FL in a dose-dependent manner and had no effect on hTERT-All (Fig. 4e, f). Down-regulation of this active hTERT transcript may explain short-term inhibition of telomerase activity.

### CX-5461 weakly decreased hTERT protein expression but effectively inhibited telomerase activity in GBM cells

**Fig. 5:**
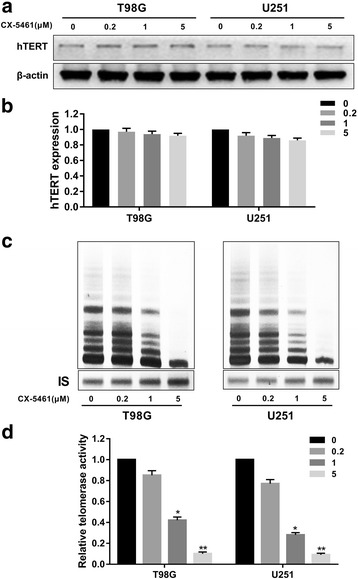
CX-5461 weakly decreased hTERT protein expression, but effectively inhibited telomerase activity in GBM cells. **a** The hTERT protein was detected both in T98G and U251 cells by immunoblotting after treatment with CX-5461 for 48 h, and β-actin was used as loading control. **b** The histogram showed hTERT protein expression levels relative to control. *P*>0.05 vs. control group. **c** After treatment with CX-5461 for 48 h, the telomerase activity of GBM cells was detected by TRAP assay. **d** The histogram showed a significant dose-dependent telomerase activity inhibition of CX-5461 in GBM cells. *P < 0.05, **P < 0.01

To better understand the potential mechanisms of CX-5461 mediated anti-telomerase activity, we performed western blot using hTERT antibody. The telomerase activity inhibition of CX-5461 was investigated by traditional TRAP assay in T98G and U251 cells. GBM cells were treated with CX-5461 for 48 h at concentrations ranging from 0 to 5 μM. The results showed that CX-5461 only weakly down-regulated hTERT protein expression in T98G and U251 cells (Fig. 5a, b). In contrast, significant dose-dependent telomerase activity inhibition was observed in GBM cells after treatment with CX-5461 (Fig. 5c, d). At 5 μM drug concentration, telomerase activity was almost completely inhibited. These results indicate that hTERT-β could be translated into a truncated hTERT protein without telomerase activity.

### CX-5461 selectively inhibited GBM cell proliferation and induced G2/M cell cycle arrest

**Fig. 6:**
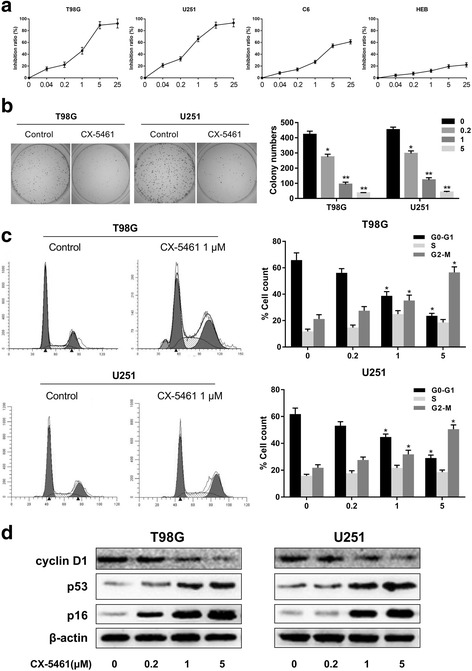
CX-5461 selectively inhibited cell proliferation and induced G2/M cell cycle arrest. **a** T98G and U251 cells exhibited significant inhibition of cell proliferation when 0.04–25 μM CX-5461 was used. C6 cells showed a modest cytotoxic effect, while human normal astrocyte HEB cells showed only a weak cytotoxic effect. **b** Representative images of colony formation and analysis of the colony numbers were observed in T98G and U251 cells after treatment with CX-5461 at indicated concentrations for 8 days. **c** G2/M cell cycle arrest was induced by CX-5461 in T98G and U251 cells. After treatment with CX-5461, cells were collected and stained with propidium iodide (PI), and then DNA content was determined by flow cytometry. **d** The cell cycle related proteins cyclin D1, p53, p16 were detected by immunoblotting after treatment with CX-5461 for 48 h, and β-actin was used as loading control. *P < 0.05, **P < 0.01 vs. control group

Significant dose-dependent growth inhibition induced by CX-5461 was observed in T98G, U251 and C6 cells (Fig. 6a). After exposure to CX-5461 (1 μM) for 48 h, significant cell inhibitory effects were found in GBM cells but not in human normal astrocyte HEB cells (Fig. 6a). Moreover, compared to the control group, colony formation was inhibited in GBM cells after 8 days of incubation with CX-5461 (Fig. 6b). After treatment with CX-5461, GBM cells were collected and stained with propidium iodide (PI), and then DNA content was determined by flow cytometry. As shown, G2/M cell cycle arrest was induced by CX-5461 in T98G and U251 cells (Fig. 6c). Furthermore, western blot results showed that CX-5461 increased the protein levels of p53 and p16, and decreased the protein levels of cyclin D1 both in T98G and U251 cells (Fig. 6d). The above results indicate that CX-5461 can selectively inhibit cell proliferation and interfere with the cell cycle progression of GBM cells with the cell cycle progression of GBM cells.

### Short-term apoptosis was evoked by CX-5461-induced DNA damage response

**Fig. 7:**
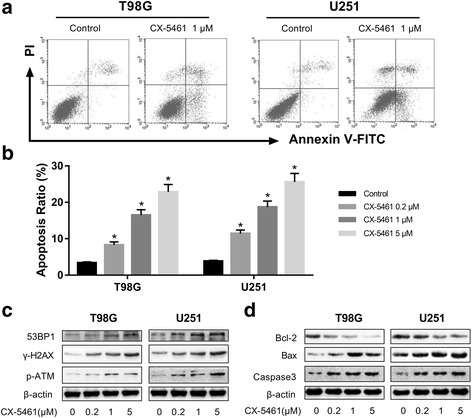
Short-term apoptosis was evoked by CX-5461-induced DNA damage response. **a, b** CX-5461 could induce apoptosis in a dose-dependent manner in T98G and U251 cells. *P < 0.05 vs. control group. **c** After treatment for 48 h, CX-5461 induced a strong increase of γ-H2AX, 53BP1, and p-ATM, which are hallmarks of DNA double-strand break response. **d** The expression of Bcl-2 was significantly decreased, meanwhile the activities of cleaved caspase-3 and Bax were markedly elevated compared with the control group

To determine the effect of CX-5461 on cell apoptosis during short-term treatment, we exposed T98G and U251 to CX-5461 in a concentration range of 0–5 μM and performed annexin V assay to assess percentage of apoptotic cells. As shown (Fig. 7a, b), CX-5461 could induce apoptosis in a dose-dependent manner in T98G and U251 cells. Next, we investigated whether the apoptosis induced by CX-5461 is related to the generation of DNA damage response. As predicted, treatment with CX-5461 for 48 h induced a strong increase of γ-H2AX, 53BP1, and p-ATM, which are hallmarks of DNA double-strand break response [38, 39] (Fig. 7c). Moreover, we examined the changes in apoptotic related proteins after treated with CX-5461 for 48 h. Compared with the control group, the expression of Bcl-2 significantly decreased, while the activity of cleaved caspase-3 and Bax significantly increased. (Fig. 7d). Based on these results, we demonstrated that apoptosis induced by CX-5461 is associated with the generation of DNA damage response. CX-5461 can not only inhibit telomerase, but also triggers a series of telomere-related cellular events to against GBM cells.

## Discussion

Numerous studies in the past three decades revealed that telomerase activation is one of the most important mechanisms in carcinogenesis [21, 40, 41]. The regulation of telomerase activity is fairly complicated, including various levels such as transcription, post-transcription, post-translation and sub-cellular localization [7]. Recent studies revealed an important role for alternative splicing, a post-transcriptional mechanism of hTERT mRNA in the regulation of telomerase activity [11, 12, 26, 42]. It has been established that there are at least five different alternative splicing sites of hTERT, among which only hTERT-FL mRNA encodes a functional hTERT protein essential for the active enzyme [10–12]. The frequent finding of hTERT transcripts in various tumor types indicates that a splicing control of hTERT mRNA for telomerase inactivation may occur in multiple human tumors.

The present data clearly demonstrated that hTERT-FL mRNA is detected in about 86.8% GBMs, 73.9% ODMs, and 42.9% OAMs. Especially, two hTERT alternative splice variants (hTERT+β and hTERT–β) were detected in human glioma tissues and GBM cell lines. Accordingly, cases with hTERT+β transcript were consistent with the expression of hTERT-FL transcript. Moreover, 89% GBMs, 71% ODMs and 46% OAMs exhibited high TA, which was consistent with the expression of hTERT-FL or hTERT+β transcript. However, most studies simply measured hTERT-All transcript or focused on telomerase activity, which greatly depends on assay conditions and sample preservation [16, 19, 43]. We suggest that analysis of hTERT-FL mRNA and telomerase activity should be completed in future related studies.

The clinical outcomes of patients with gliomas show great differences. Although extensive efforts have been made to determine the exact biomarkers for gliomas, more reliable prognostic predictors are required [19, 20]. It has been shown that telomerase activity and hTERT-FL expression are powerful predictors for neuroblastomas [18, 44] and certain types of malignancies [15, 17, 42, 45, 46]. When hTERT+β transcript expression or high TA was correlated with the clinical variables of the patients, its presence is found to be significantly correlated with age ≥ 50, KPS ≥ 75, tumor size ≥6 cm, and higher tumor WHO grades. Survival analysis showed a significant survival benefit for patients lacking hTERT+β or TA in different WHO grade gliomas. Hence, our results demonstrate a correlation between clinical outcome and hTERT-FL transcript or higher TA. It is interesting to define whether these two variables will independently add prognostic information in larger cohorts of patients with gliomas. In particular, grade II and III telomerase-negative ODMs and OAMs are frequently ALT-positive [47, 48]. Unfortunately, we did not detect ALT in glioma samples, which is a disadvantage of this study. For telomerase-negative patients, ALT may also have some effect on prognosis.

Although hTERT alternative transcripts have been described in a number of malignant tumors, little is known about the causes of splice variants and how they are regulated [27]. A previous study showed that TGF-β1 converts splicing from hTERT-FL to hTERT–β transcript in epidermal cells [29]. The antisense oligonucleotides were reported to change the splicing patterns of hTERT pre-mRNA to induce telomerase inhibition and cell growth decline in human prostate cancer cells [28]. Another study reported a G-quadruplex stabilizer named 12,459 switching splicing from hTERT-FL to hTERT–β transcript in A549 cells [30]. G-quadruplex structures have been extensively studied in the telomeric single-stranded overhang or c-myc gene promotor sequence, and hTERT intron 6 was reported to have two G-rich sequences able to form G-quadruplex, which may affect β splicing [30–32]. Similarly, CX-5461 also promotes the stabilization of these G-quadruplexes, as demonstrated by specific PCR-stop assay [35]. Our current data indicate that CX-5461 can modify alternative splicing of hTERT, convert hTERT-FL to hTERT-β transcript, and hardly affect hTERT-All transcription in T98G and U251 cells. A possible explanation for the hTERT-β splicing would be that these G-rich sequences of intron 6 bind to a splicing factor involved in the negative control of hTERT-β alternative splicing. In that case, the stabilization of this region into G-quadruplex by CX-5461 would impair the binding of this factor and would provoke hTERT-β alternative splicing.

We also demonstrated that CX-5461 could effectively inhibit telomerase activity independent of hTERT protein expression in GBM cells. It is consistent with the results of a study in which TERT-β transcript can be translated into a truncated hTERT protein without telomerase activity [49]. The effect of CX-5461 resulted in downregulation of hTERT+β transcript and increase in hTERT-β transcript, explaining the downregulation of hTERT-FL and telomerase activity. A detailed analysis of the action of CX-5461 aiming to reconstitute the splicing of hTERT would be necessary to understand the biological effect of this stabilizer.

CX-546 was reported to have biological efficacy in multiple studies [33–35]. In this study, we demonstrated that CX-5461 could selectively inhibit GBM cell proliferation and induce G2/M cell cycle arrest. This result is consistent with the periodic arrest induced by most telomere G-quadruplex ligands, and there is evidence that the response of telomere injury is G1 or G2 cell cycle arrest [37]. Apoptosis in cancer cells induced by DNA damage response has been described as one of the characteristics of G-quadruplex ligands, and our previous study has demonstrated it [34–37]. This study shows that short-term apoptosis induced by CX-5461 is associated with the generation of DNA damage response. CX-5461 can not only inhibit telomerase activity, but also triggers a series of telomere-related cellular events to against GBM cells. Unlike other G-quadruplex ligands, CX-5461 may contribute to inhibition of telomerase activity through two different mechanisms. In addition to the stabilization of G-quadruplex in telomeric overhang repeats as reported, CX-5461 could also induce downregulation of active hTERT transcript to inhibit telomerase activity.

## Conclusions

In conclusion, our study clarifies the patterns of alternative splice variant of hTERT and highlights the striking correlation between hTERT+β expression and telomerase activity in gliomas. The alternative splicing of hTERT is verified to be correlated with histopathological and clinical parameters of gliomas, and could serve as a prognostic marker or possibly therapeutic target for glioma patients. However, the exactly regulatory mechanisms and biological functions of hTERT alternative splicing are still uncertain. Our results also suggest that G-quadruplex stabilizer CX-5461 can regulate the splicing patterns of hTERT and inhibit telomerase activity in GBM cells. In addition, CX-5461 has cytotoxicity to GBM cells and can cause telomere DNA damage response, induce G2/M arrest and cell apoptosis.

## Abbreviations

GBM: Glioblastoma
hTERT: Human telomerase reverse transcriptase
hTERT-All: All the variants of hTERT;
hTERT-FL: Full-length hTERT
OAM: Oligoastrocytoma;
ODM: Oligodendroglioma
TA: Telomerase activity;
TRAP: Telomere repeat amplification protocol
